# Self-Assembled Peptide Nanostructures for ECM Biomimicry

**DOI:** 10.3390/nano12132147

**Published:** 2022-06-22

**Authors:** Davide Marin, Silvia Marchesan

**Affiliations:** Chemical and Pharmaceutical Sciences Department, University of Trieste, 34127 Trieste, Italy; davide.marin@studenti.units.it

**Keywords:** self-assembly, peptides, proteins, RGD, collagen, ECM, hydrogels, biomaterials, biomimicry, nanofibrils

## Abstract

Proteins are functional building blocks of living organisms that exert a wide variety of functions, but their synthesis and industrial production can be cumbersome and expensive. By contrast, short peptides are very convenient to prepare at a low cost on a large scale, and their self-assembly into nanostructures and gels is a popular avenue for protein biomimicry. In this Review, we will analyze the last 5-year progress on the incorporation of bioactive motifs into self-assembling peptides to mimic functional proteins of the extracellular matrix (ECM) and guide cell fate inside hydrogel scaffolds.

## 1. Introduction

Proteins exert a wide variety of functions in cells and living organisms and can be considered one of the key building blocks of life. It is thus not surprising that they are very popular components in the development of new solutions as biomaterials for applications in medicine [1,2,3,4], cosmetics [5,6] and food science [7], including highly biotechnological products, such as cultured meat [8]. Collagen [9,10], gelatin [11,12,13,14], keratin [15,16,17], and silk fibroin [18,19] are amongst the most widely used proteins to develop biomaterials.

However, proteins present also limitations, such as low oral bioavailability, for which new nanotechnological carriers are continuously being developed [20]. Furthermore, their production on a large scale can be costly, inefficient, and present batch-to-batch high variability, also in terms of purity, correct folding, and, thus, activity. For all these reasons, new nanotechnological alternatives for protein biomimicry are highly sought after, to reduce costs and increase efficiency both for the production process and for the final product performance and lifetime [21]. Amongst the various substitutes for their mimicry, short peptide sequences based on bioactive motifs certainly play an elected role. Peptide-protein interactions are indeed crucial for the design of biomaterials [22]. In particular, the use of minimalistic bioactive motifs to this end was exhaustively reviewed in 2017 [23], and for this reason, here we will cover the recent progress in the field made since then. 

## 2. Self-Assembling Short Peptides with Bioactive Motifs for Hydrogel Biomaterials

Over the last three decades, great efforts have been devoted to the design of self-assembling peptides to attain nanostructured biomaterial gels. Readers interested in the details of their various types of design are recommended to read a recent book chapter that provides a comprehensive overview of the topic [24]. Several recent reviews also cover this area [25,26], as well as the use of enzymes to control self-assembly [27], applications for the delivery of drugs and therapeutics [28,29,30,31,32,33], proteins [34,35], and, more generally biomedical uses [36], with a specific focus on antimicrobials [37], cancer [38], and wound healing too [39].

**Figure 1:**
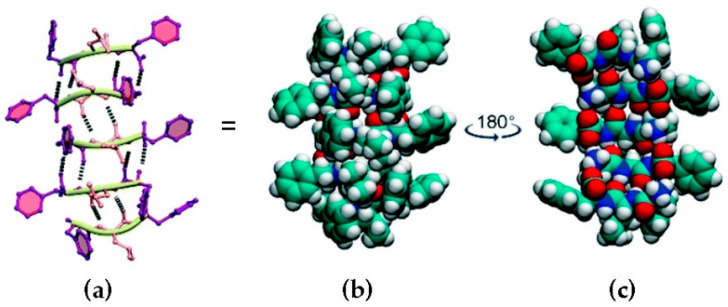
Heterochiral peptide self-assembly. l-Phe-d-Leu-l-Phe forms stacks held together by H-bonding between amides (**a**), and space-fill representations (**b**,**c**) show the amphipathic character of the stacks, which display a hydrophobic face with the peptide sidechains (**b**) and a hydrophilic face with amide bonds (**c**). Carbon atoms are shown in green, hydrogen in white, nitrogen in blue, and oxygen in red. Reproduced from [45].

Briefly, the vast majority of peptides for self-assembly into hydrogels are amphipathic in nature. Self-assembly in water is hydrophobically driven, and aromatic components play an elected role in the stabilization of steric zippers and hydrophobic interactions that hold together the peptide superstructures. Hydrophilic components are crucial to ensure good water solubility and hydrogelation ability, and often are involved in weak interactions, such as H-bonding, binding peptides together [24]. Popular approaches for their design use complementary charges alternated with hydrophobic amino acids [40,41], polyaromatic N-caps [42], peptide amphiphiles (PA) [43], or heterochirality [44], to attain an amphipathic character for correct self-assembly and hydrogelation (Figure 1).

Considering that the shorter the motif, the lower and easier will be the cost of production, it is thus not surprising that ultra-short peptides are amongst the most attractive supramolecular peptide gelators [46]. In particular, cyclic [47,48,49] or linear dipeptides [50,51,52,53,54,55] are amongst the simplest options as building blocks for hydrogels, however, tripeptides are better positioned for bioactivity. Indeed, it was shown that 25 atoms other than hydrogen constitute the ideal size for drugs and drug-like molecules for maximal ligand-binding efficacy and bioactivity, and this number corresponds to the average number for a tripeptide [56]. Indeed, there are several tripeptides, or slightly longer sequences, that are used as bioactive motifs for protein mimicry in nanostructured hydrogel biomaterials [23], and the examples reported in the last 5 years are summarized in Table 1.

In particular, self-assembling peptides that form nanofibrillar hydrogels are ideally suited to mimic the extracellular matrix (ECM), which comprises a structurally similar nanofibrous network. The ECM is composed of a complex and dynamic mixture of proteins (e.g., laminin, fibronectin, collagen), glycosaminoglycans (e.g., hyaluronic acid and heparin), and growth factors, that altogether allow a continuous remodeling to respond to the requirements of resident cells to sustain their growth. It is thus not surprising that peptide-based nanofibrous hydrogels are often used in regenerative medicine as ECM mimics, as described in the examples below, to foster cell differentiation and proliferation and facilitate tissue-repair processes [57].

**Table 1:** Latest reported supramolecular peptide-based hydrogels with bioactive motifs.

Bioactive Sequence	Gelator	Function	Model	Ref.
RGD(S) and mimic	KFE-RGD KFE-RDG KFE-8	Cell adhesion	hMSC	[58]
	RADA16	Cell adhesion	3T3 cells	[59]
	G-Y sequence	Cell adhesion	L929 cells	[60]
	RGDSGAITIGC	Cell proliferation	3T3 cells	[61]
	E_3_-PA E_3_G_3_Ada-PA	Cell adhesion	3T3 cells	[62]
	Fmoc-FF Fmoc-RGD	Cell adhesion and Differentiation	3A6 cells Mice	[63]
	Fmoc-FF Fmoc-RGD	Cell delivery	Osteoblast Fibroblast Mice	[64]
	Fmoc-F_5_-Phe Fmoc-K(Fmoc)-RGD	Antimicrobial Cell adhesion	3T3 cells	[65]
	Silk fibroin Nap-FFRGD	Cell adhesion Angiogenesis	HUVEC Mice	[66]
	Silk fibroin Nap-FFRGD	Cell adhesion Osteogenesis	mBMSC Mice	[67]
	Collagen-like peptide	Neuronal cell maturation	Neuronal-glial cells	[68]
	Fmoc-FFβAR(K)βA-OH Fmoc-FFβAR(K)βA-NH_2_	Cell adhesion	MSC-P5, N2a, A549 cells	[69]
	Fmoc-FFGGRGD	Inhibition of β_1_-integrin, FAK and Akt expression	Tenon’s capsule fibroblasts	[70]
	Fmoc-FRGDF Agarose	Laminin and fibronectin mimic	-	[71]
	C_16_-V_3_A_3_E_3_E_3_RGDS C_16_-V_3_A_3_K_3_SVVYGLR C_16_-V_3_E_3_DGEA	Osteogenesis and angiogenesis	hAMSC, HUVEC	[72]
	Fmoc-FRGDF Fmoc-PHSRN	Cell adhesion	HMFC	[73]
	E_1_Y_9_-ALK E_1_Y_9_-RGDS E_1_Y_9_-DGR E_1_Y_9_-PRG	Osteogenesis	MC3T3-E1 cells	[74]
LDV	fFL and fFLDV	Cell adhesion	L929 cells	[75]
PHSRN	Fmoc-FRGDF Fmoc-PHSRN	Cell adhesion	HMFC	[73]
IKVAV	RADA4GGSIKVAV	Neuronal stem-cell delivery Anti-inflammatory	hMgSC Mice	[76]
	Fmoc-DIKVAV	Neuronal cell differentiation	Mice	[77]
	Fmoc-DDIKVAV	Neuronal cell differentiation	hPSC Mice	[78]
	IKVAV-PA	Neuronal cell differentiation	hESC Mice Human temporal bone	[79]
	IKVAV-PA	Neuronal cell differentiation	BMSC	[80]
	IKVAV-PA YRSRKYSSWYVALKR	Spinal cord injury repair (laminin and FGF2 mimicry)	Mice	[81]
	Fmoc-DIKVAV Agarose	Laminin and fibronectin mimic	-	[71]
	Fmoc-IKVAV Fmoc-YIGSR	Neuronal cell growth	C6 cells SHSY5Y cells	[82]
YIGSR	KLD-IKVAV KLD-YIGSR	Vasculogenesis	HUVEC, hMS cells	[83]
	Nap-GFF(p)YIGSR	Anticancer Self-assembly directly on cells	HeLa cells	[84]
	Fmoc-IKVAVFmoc-YIGSR	Neuronal cell growth	C6 cells SHSY5Y cells	[82]
YSV	FKFEYYSV	Anticancer	A549 cancer cells	[85]
	Taxol-EYSV	Anticancer	HeLa, A2780 cells Mice	[86]
	Nap-GffyGYSV	Anticancer	BEL-7402, HeLa, MCF-7 cells Mice	[87]
	Nap-Gff(p)YSV	Anticancer Self-assembly directly on cells	HeLa, A549 cells	[88]
	Nap-GFF(p)YSV	Anticancer Self-assembly directly on cells	HeLa cells	[84]
HAV	Fmoc/Nap-HAVDI	Cell adhesion	C6, L929 cells	[89]
	HAV-PA E-PA	Chondrogenesis	rMSC	[90]
	KLD-12	Chondrogenesis	hMSC	[91]
SVVYGLR	RADA16	Angiogenesis	HCN-A94-2 cells Zebrafish	[92]
	C16-V_3_A_3_E_3_E_3_RGDS C16-V_3_A_3_K_3_SVVYGLR C16-V_3_E_3_DGEA	Osteogenesis and angiogenesis	hAMSC, HUVEC	[72]
DGEA	C16-V_3_A_3_E_3_E_3_RGDS C16-V_3_A_3_K_3_SVVYGLR C16-V_3_E_3_DGEA	Osteogenesis and angiogenesis	hAMSC, HUVEC	[72]
KTT	C_16_KTTβAH	Collagen production	MCF-7, MDA-MB-231, HDFa cells	[93]
βAH	C_16_KTTβAH	Anticancer	MCF-7, MDA-MB-231, HDFa cells	[93]
ALKRQGRTLYGF	E1Y9-ALK E1Y9-RGDS E1Y9-DGR E1Y9-PRG	Osteogenesis	MC3T3-E1 cells	[74]
DGRDSVAYG	E1Y9-ALK E1Y9-RGDS E1Y9-DGR E1Y9-PRG	Osteogenesis	MC3T3-E1 cells	[74]
PRGDSGYRGDS	E1Y9-ALK E1Y9-RGDS E1Y9-DGR E1Y9-PRG	Osteogenesis	MC3T3-E1 cells	[74]

### 2.1. RGD

The RGD motif is by far the most widely applied bioactive motif in biomaterials science. It originates from fibronectin and it is well known to promote cell adhesion because of its affinity for integrins expressed on cells’ membranes, and hydrogels containing the RGD motifs can mimic the extracellular matrix (ECM) [94]. For this reason, the RGD motif is studied as a scaffold for cell cultures, or for biomedical devices and drug delivery. As the α_IIb_β_3_ integrin is expressed on platelets’ surfaces, RGD was also considered an antithrombotic drug. However, due to its peptidic nature, RGD is easily degraded in biological environments, thus prompting research to move towards the design of RGD mimetics [95].

Mechanical and adhesion properties of hydrogels based on the self-assembly of RGD-modified peptides can be tuned by varying the hydrogel composition. Recently, a hydrogel was obtained using the gelator peptide KFE-8 (i.e., Ac-FKFEFKFE-NH_2_) modified with either the bioactive RGD sequence (i.e., Ac-GRGDSPGGFKFEFKFE-NH_2_) or the inactive, scrambled RDG sequence (i.e., Ac-GRDGSPGGFKFEFKFE-NH_2_) [58]. The adhesion properties of this hydrogel could be modified by varying the composition in terms of active KFE-RGD and inactive KFE-RDG whilst keeping constant the total concentration of both peptides, thus constant mechanical properties. Conversely, increasing the concentration of the gelator KFE-8 resulted in an increase of gel stiffness. In this manner, it was possible to tailor the differentiation of human mesenchymal stem cells, with adipogenesis being favored by softer gels with lower concentrations of RGD. In another recent work, RGD-modified hydrogels were obtained by simply changing an alanine with glycine in different positions of the well-known gelator peptide RADA16 [59]. This study showed that the substitution position has a great impact on the gelation ability and properties of the system, as well as on the cell adhesiveness. In particular, the substitution A6G inhibited β-sheet formation, whilst A10G and A14G resulted in twisted molecular alignment along with the sheets, and overall higher viscoelasticity and bioadhesiveness of the resulting gels. A similar approach was reported using the R-Y peptide sequence (i.e., RRKSYSGILGDLIQAVIRYY) from cp-52k to form a hydrogel and inserting the RGDS sequence in different positions of the chain [60]. In this case, too, it was demonstrated that the position of the RGDS sequence influenced the ability of R-Y to form β-sheets and, thus, the mechanical properties of the hydrogel. Specifically, the introduction of the RGD motif at the N-terminus resulted in higher β-sheet content, while the secondary conformation was not significantly changed upon inclusion of RGD in the middle of the sequence, and was reduced when positioned at the C-terminus, with consequently reduced gelation ability.

Recently, the self-assembling RGDSGAITIGC sequence containing the RGD motif was discovered via computational methods and tested for fibroblast proliferation [61]. This sequence preserved the ability to form a hydrogel with β-sheet amyloid structure and carried the RGD adhesion motif at one end, while at the other end there was a cysteine for further functionalization with other bioactive molecules or binding to metal surfaces.

A non-covalent approach via host-guest interactions was reported too [62]. Briefly, a hydrogel based on PA and PA functionalized with adamantane (ada) was formed, and host-guest interactions occurred between ada and a β-cyclodextrin (βCD) functionalized with the RGDS peptide. This hydrogel was studied for fibroblasts adhesion and growth, and it was observed that the host-guest interaction was critical in the epitope presentation, as control hydrogels without ada or without βCD showed a cell morphology and spreading comparable to the PA hydrogel alone.

The Fmoc protecting group is an established promoter of short peptide self-assembly via π-π stacking interactions, as is the FF sequence for amyloid proteins. Furthermore, the Fmoc-F sequence is also known to display antibacterial properties [96]. A hydrogel based on Fmoc-FF and Fmoc-RGD was developed to induce mesenchymal stem cells’ proliferation and to enhance their induced differentiation compared to the Fmoc-RGE non-bioactive hydrogel [63]. A similar injectable hydrogel based on Fmoc-FF and Fmoc-RGD was enriched with magnetic nanoparticles in order to increase the mechanical properties of the gel and also to allow for a magnetically targeted cell delivery for tissue regeneration [64]. The Fmoc-based self-assembly strategy allowed to obtain a hydrogel based on Fmoc-F_5_-Phe and a Fmoc-RGD derivative suitable for cell cultures, due to the adhesion properties of RGD combined with antimicrobial properties of Fmoc-F_5_-Phe [65]. Interestingly, fluorescence measurements allowed to establish the cooperative co-assembly of the different peptide sequences.

**Figure 2:**
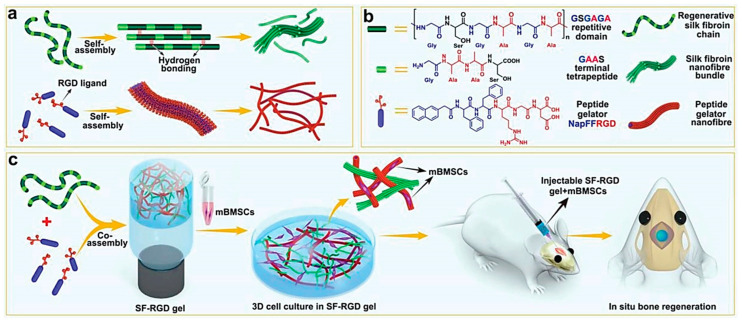
(**a**,**b**) Molecular structures and self-assembling properties of peptide gelator (Nap-FFRGD) and silk fibroin (SF) for the formation of nanofiber and nanofibril bundle structures individually; (**c**) illustration of the preparation process for SF-RGD gel from Nap-FFRGD and SF, and its biological functions to enhance osteogenesis of encapsulated mBMSCs for bone regeneration in calvarial defect areas of mice. Reprinted with permission from [67], © 2022 WILEY-VCH Verlag GmbH & Co. KGaA, Weinheim, Germany.

Another gelation strategy is the functionalization of peptides with the naphthyl group (Nap) that can perform π-π stacking interactions and lead to self-assembly [42]. In two recent works, this strategy was used to obtain hydrogels based on silk fibroin (SF) and Nap-FFRGD that were studied as scaffolds for regenerative medicine to promote angiogenesis [66] and osteogenesis [67] (Figure 2). The presence of the Nap-FFRGD gelator was key to obtaining gels at lower concentrations of SF. In another work, a PEGylated collagen-like peptide functionalized with the RGD motif (PEG-CLP-RGD) allowed for better maturation and structural organization of neuronal cells, relative to controls with PEG-CLP hydrogel or poly-l-lysine [68]. Finally, a hybrid polysaccharide-peptide hydrogel based on the co-assembly of agarose and Fmoc-FRGDF was found to better mimic the physical and chemical properties of ECM compared to hydrogels based on the peptide alone [71]. Indeed, the ECM matrix is rich in proteoglycans, besides fibrous proteins, thus the inclusion of polysaccharides offers better biomimicry of the natural scaffold.

A problem in the use of peptide-based systems for biological applications is their rapid enzymatic degradation. This problem can be solved using β- or d-amino acids or designing peptidomimetics. A recent work reported the preparation of thixotropic hydrogels based on Fmoc-FFβAR(K)βA-OH peptide and the amidated Fmoc-FFβAR(K)βA-NH_2_ containing the RGD-mimetic R(K) motif. Both hydrogels were tested on various types of cells (including neuronal cells) with good results in terms of cell adhesion and growth, and the amidated peptide also showed antimicrobial activity suitable for cell cultures [69].

Finally, a Fmoc-FFGGRGD-based hydrogel proved that the RGD sequence also interferes with gene expression in Tenon’s capsule fibroblasts, in particular reducing the β_1_-integrin, FAK, and Akt expression in order to inhibit fibrogenesis and scar formation that limits the success of glaucoma filtration surgery [70].

### 2.2. LDV

**Figure 3:**
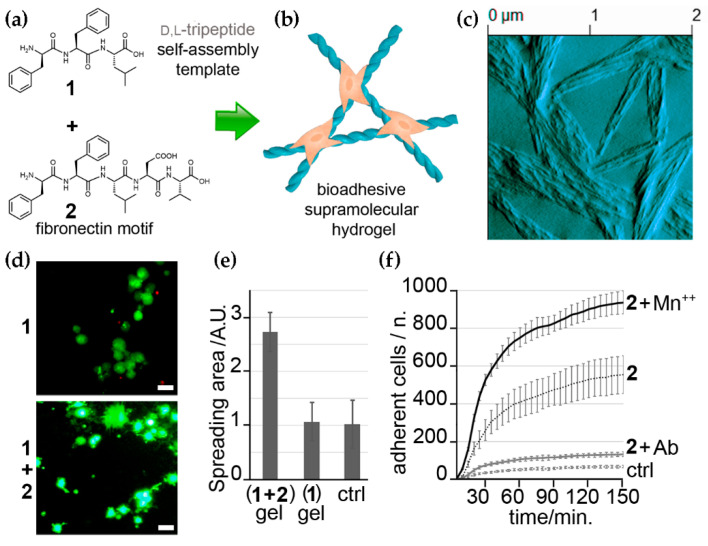
(**a**) Chemical structures of two self-assembling short peptides with structural (1) and bioactive (2) roles for the co-assembly into bioadhesive hydrogels (**b**). (**c**) AFM image of the co-assembled gel. (**d**) live-dead cell-microscopy images of the control (1) and the bioadhesive (1 + 2) hydrogels with fibroblasts. (**e**) Quantification of cell spreading. (**f**) Adherent cells’ count in the presence of Mn^++^ and a β_1_ integrin-blocking antibody (Ab) demonstrates integrin engagement for cell adhesion on the biomaterial and successful ECM mimicry. Adapted from [75].

LDV is another fibronectin-derived tripeptide discovered in 1991 as an activator of β_1_ integrins and a cell adhesion promoter [97,98]. It was also discovered to have anti-inflammatory [99] and anti-metastatic activities [100]. The first incorporation of this bioactive tripeptide in a supramolecular peptide hydrogel was reported by our group using the self-assembling d-Phe-l-Phe-l-Leu (i.e., fFL) together with the bioactive fFLDV [75]. The so-obtained hydrogel successfully acted as a scaffold for cell adhesion and spreading, and the active engagement of integrins was demonstrated (Figure 3). In this case, the choice for LDV over RGD was determined also by the increased hydrophobicity of the former sequence, in order to avoid possible hindrance of the hydrophobically-driven peptide self-assembly in water that enables hydrogelation.

### 2.3. PHSRN

PHSRN is another fibronectin-derived bioactive motif, which acts in synergy with RGD for the binding of β_1_ integrins and was found to accelerate cell invasion and wound healing [101,102,103]. However, most researches focus only on RGD because the synergistic effect requires a precise spatial disposition of the two bioactive fragments, and so in most cases, the combination of RGD and PHSRN does not show significant improvements in cell adhesion [104,105]. Recently, a hydrogel based on Fmoc-FRGDF and Fmoc-PHSRN was successfully obtained, with increased cell adhesion compared to the Fmoc-FRGDF peptide alone [73]. It was also noted that, while Fmoc-FRGDF alone could self-assemble, Fmoc-PHSRN alone could not, and Fmoc-FRGDFPHSRN combined peptide gave a weak gel. This was due to the disruptive presence of the rigid proline, so mixing the two Fmoc-peptides was necessary to get a hydrogel with synergistic bioadhesiveness. Indeed, proline is well-known for its β-breaker role, which can be exploited to modulate the self-assembling behavior of short-peptide gelators based on the β-sheet prone FF motif [106].

### 2.4. IKVAV

IKVAV is another integrin-binding peptide that originates from laminin, so that it mimics the ECM, it promotes cell adhesion and growth, and differentiation of stem cells too [107,108]. For these reasons, IKVAV-containing peptides are widely studied to yield cell cultures and medical scaffolds for regenerative medicine. One of the hardest challenges in this field is to repair nerve tissues after damage to the peripheral [109] or central systems [110]. The IKVAV motif plays an elected role to this end, thanks to its favorable interactions with neurons and stem cells [111].

In a recent work, a hydrogel-based on the self-assembling and bioactive RADA4GGSIKVAV peptide was tested on mice [76]. It led to good results in improving brain injuries via neuronal stem cell delivery and it was also found to inhibit the molecular inflammatory pathway. Similar results were obtained with Fmoc-DIKVAV-based hydrogels used to treat Parkinson’s in a mouse model [77]. In this case, the hydrogel was loaded with a glial cell line-derived neurotrophic factor to induce the differentiation of both delivered and endogenous stem cells. This scaffold showed a good ability in enhancing neural-cell survival and differentiation, as well as in inhibiting the formation of glial scars. In another recent work, a hydrogel based on the very similar Fmoc-DDIKVAV was used to treat stroke-affected mice, resulting in the recovery of motor function, thanks to the enhanced cell differentiation, growth, and incorporation [78]. The reason for choosing the sequence with the additional two aspartic acid residues was to modulate the self-assembly behavior and obtain hydrogels at the physiological pH of 7.4 [78].

PA-based hydrogels with incorporated IKVAV sequences were successfully used to create a niche in mice inner ear to increase the survival and support the differentiation of neuron progenitors to regenerate the spiral ganglion [79]. While in all other previous cases the differentiation of stem cells was externally induced, and only supported and enhanced by IKVAV, a recent work reported the successful differentiation of BMSCs induced only by this peptide sequence contained in a PA-based hydrogel [80]. Remarkably, the combination of IKVAV-PA with another PA functionalized with an FGF-2 mimetic sequence, allowed for locomotor recovery after spinal cord injury in a mouse model [81]. Indeed, the use of more than one biomolecule is a promising strategy for regeneration, as demonstrated for the co-assembly of Fmoc-DIKVAV and agarose [71], as well as for the concomitant use of two laminin-derived motifs in Fmoc-IKVAV and Fmoc-YIGSR [82].

### 2.5. YIGSR

YIGSR is another cell adhesion domain found in the ECM protein laminin [112,113], and it is often used in combination with other peptides. A recent work reported the formation of a supramolecular hydrogel based on the self-assembling KLD peptide (i.e., KLDLKLDLKLDL) elongated with the bioactive YIGSR sequence (i.e., KLDLKLDLKLDLYIGSR) [83]. This hydrogel showed better vascularization ability on hMSC/HUVEC cell cultures, than the analogue KLD-IKVAV (i.e., KLDLKLDLKLDL-IKVAV) hydrogel.

The enzyme-instructed self-assembly (EISA) strategy was also applied to YIGSR-containing peptides, to form a self-assembled hydrogel directly on HeLa cells overexpressing alkaline phosphatase (ALP), so that a phosphorylated gelator precursor (that was too hydrophilic to self-assemble) could be dephosphorylated in situ and gel [84]. Importantly, direct use of the non-phosphorylated Nap-GFFYIGSR was not effective due to its poor solubility in water, thus confirming the effectiveness of the EISA approach. The co-assembly of Fmoc-YIGSR and Fmoc-IKVAV, both cell adhesion segments from laminin, produced a supramolecular hydrogel that displayed a synergistic effect in controlling neuronal cell adhesion and growth [82].

### 2.6. YSV

YSV is a bioactive tripeptide that attracted considerable attention in the field of drug discovery due to its anticancer properties against various types of cancer. The action mechanism involves the inhibition of both P-glycoprotein and histone deacetylase [114]. However, a millimolar concentration of peptide is needed to see any effect, and its peptidic nature limits its bioavailability. For this reason, different approaches are needed in order to enhance its anticancer effect and use this peptide as a drug, including the use of d-amino acids and the formation of hydrogels that also allow for the simultaneous delivery of other anticancer drugs, for a synergic treatment to overcome drug resistance. It was previously demonstrated that the formation of hydrogels on cancer cells via self-assembly of d-peptides interrupts intercellular exchanges leading to apoptosis, and that the EISA approach is applicable as phosphatases can dephosphorylate d-peptides too (e.g., Nap-ffy phosphorylated on y) [115].

For example, a peptide-sequence screening for hydrogelation yielded the octapeptide FKFEYYSV, composed of the bioactive tripeptide YSV and the gelation moiety FKFEY which also increased the anticancer activity of YSV. In addition, hydroxycamptothecin was loaded on the hydrogel for simultaneous drug delivery, and the whole system showed good anticancer activity, without toxicity for non-cancerous cells [85]. A similar combined approach was developed using an EYSV peptide linked to taxol (taxol-EYSV) [86]. In this case, the hydrogel formation was driven by the auto-hydrolysis of taxol-EYSV in biological environments that led to the co-assembly of the resulting two components, showing good anticancer properties also in vivo in mice.

Another approach consisted of the use of Nap-GffyGYSV peptide, containing the Nap gelation moiety and d-amino acids to increase the gelation ability and resistance of the gel in biological environments [87]. A smarter strategy using the similar peptides Nap-Gff(p)YSV [88] and Nap-GFF(p)YSV [84] with a phosphorylated tyrosine was developed to selectively form a hydrogel via dephosphorylation of tyrosine with EISA, directly on cancer cells overexpressing alkaline phosphatase.

### 2.7. HAV

HAV is a tripeptide that originates from cadherins, which are calcium-dependent glycoproteins expressed on cell membranes and involved in cell-to-cell adhesion, cell differentiation, morphogenesis, and many other processes [116,117,118]. This motif has cell recognition and adhesion functions in cadherins [119], but also acts as a cadherin antagonist, as it binds tyrosine-kinase receptors but it is too small to cause the receptor dimerization [120].

**Figure 4:**
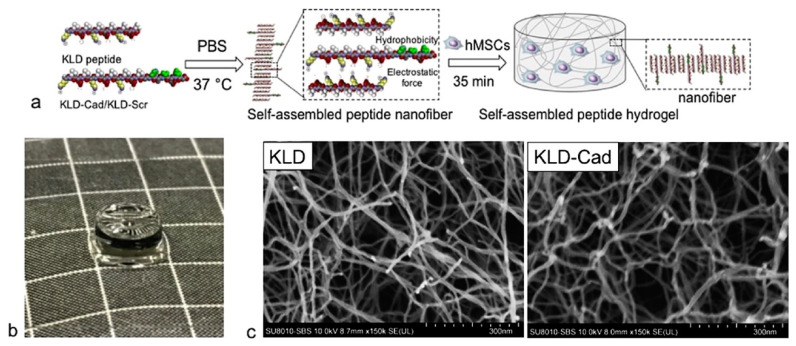
(**a**) Scheme of self-assembly of hMSC-encapsulated KLD-Cad/KLD-Scr hydrogels. (**b**) Photograph of KLD-Cad self-assembled hydrogel (d = 5 mm, h = 2.2 mm) (**c**) SEM images of KLD (left) and KLD-Cad (right) hydrogels after critical point drying show that the average diameter of self-assembled fiber in the KLD and KLD-Cad hydrogels are approximately 17.6 nm and 20.4 nm, respectively. Reprinted from [91], copyright © 2022, with permission from Elsevier.

In a recent work, the bioactive peptide HAVDI was linked to Fmoc and Nap gelling moieties [89]. In both cases, an ECM-mimic hydrogel was obtained with good cell adhesion, viability, and proliferation, and promoted normal cellular functions in both neuronal and non-neuronal cells. The use of the aromatic N-caps was key to enabling gelation, as it allowed to significantly increase the hydrophobicity (C logP ≥ 1.4) of the peptide sequence relative to the uncapped analog (C logP −3.9) that was too hydrophilic to gel. The chondrogenic properties of HAV were also proven using a hydrogel based on oppositely charged HAV-PA and E-PA [90]. Differentiation of mesenchymal stem cells into chondrocytes was artificially induced, but the hydrogel bioactive support enhanced the differentiation and organization of cells stimulating the expression of cartilage-specific markers. In another similar work, in which an HAV-modified KLD-12 self-assembling peptide was used to obtain a hydrogel, it was demonstrated that the chondrogenic abilities of this kind of support involved the inhibition of canonical Wnt/β-catenin signaling. This gel was used for the successful encapsulation of stem cells (Figure 4) [91].

### 2.8. SVVYGLR

SVVYGLR is a bioactive peptide motif derived from osteopontin that shows high avidity for α_9_β_1_ and α_4_β_1_ integrins, promoting endothelial cells adhesion and migration, and angiogenesis, with subsequently enhanced neurogenesis too. A recent work reported the formation of a hydrogel based on the RADA16 gelator peptide linked to SVVYGLR bioactive motif (i.e., Ac-RADARADARADARADASVVYGLR-NH_2_) [92]. This novel hydrogel scaffold showed excellent angiogenesis and neurogenesis in vitro and in vivo when tested on zebrafish, and could potentially act as a bioactive scaffold for the regeneration of damaged brain tissues. Furthermore, the viscoelastic properties could be tuned with peptide concentration, as the elastic modulus G’ could be increased 15–20 times as the amount of gelator increased from 1% *w*/*v* to 2% *w*/*v*.

### 2.9. DGEA

DGEA is a collagen-I mimetic motif that showed to promote adhesion and osteogenic differentiation of stem cells [121]. A combined approach involving PAs containing SVVYGLR, RGDSm, and DGEA sequences was recently reported to create a hydrogel scaffold for the formation of vascularized bone-like constructs in vitro [72]. Co-assembly was favored by electrostatic interactions between oppositely charged sequences, for which the zeta potential was indeed confirmed to be positive for the former, and negative for the latter two. The gels, which also contained tyramine-functionalized hyaluronic acid for better ECM biomimicry, could be attained with elastic moduli in the range of 0.6–3.2 kPa upon inclusion of Ca^++^ salts to favor crosslinking and gelation.

### 2.10. βAH and KTT

Carnosine is a dipeptide (βAH) with various biological functions [122,123], especially as an antioxidant due to the presence of β-alanine. It has also been proposed as a treatment for Alzheimer’s disease [124], and it was shown to have anticancer properties [125]. A recent work reported a dual-component hydrogel based on the lipopeptide C_16_KTTβAH, that displayed anticancer activity against breast cancer, and lower toxicity on non-cancerous cells, thanks to the presence of the KTT motif [93]. The KTT motif is derived from procollagen I and it is widely used in cosmetic products due to its ability to induce collagen production [126]. Interestingly, the cytotoxicity of C_16_KTTβAH was manifest also at concentrations below the critical aggregation concentration, thus suggesting that it was not related to the nanofibrillation [93]. Furthermore, this gelator displayed promising potential also in terms of biomaterial viscoelastic properties, which could be fine-tuned over a wide range (e.g., G’ from 1 kPa to 1 MPa), depending on the gelation protocol and ionic strength used.

### 2.11. Other Bioactive Motifs Combined for Osteogenesis

Finally, a hydrogel based on various bioactive peptides was reported and acted as a potential scaffold for bone regeneration due to its adhesion properties and differentiation support toward MC3T3-E1 cells [74]. In particular, the hydrogel was based on the E1Y9 peptide (Ac-EYEYKYEYKY-NH_2_), which self-assembled in the presence of Ca^2+^ ions. This was functionalized with RGDS as a cell adhesion motif, ALK (ALKRQGRTLYGF) as an osteogenic growth peptide [127], DGR (DGRDSVAYG) as a cell adhesion motif of osteopontin that is involved in many bioactivities of osteoblasts and osteoclasts [128,129], and PRG (PRGDSGYRGDS) as a cell adhesion motif from type IV collagen that promotes adhesion of osteoblast cells [130]. The gelation ability of Y1E9 relies on a β-sheet conformation, which is partially disrupted by the introduction of positive charges, as in the bioactive motifs. Despite the peptides’ ability to respond to the introduction of CaCl_2_ as a self-assembly trigger, nanofibrillation could not always be rescued, as in the case of the cationic peptide sequence with the ALK motif. This study provided further evidence that the mere introduction of bioactive sequences to self-assembling peptides requires careful design, as it can hinder peptide conformation and gelation ability, depending on the sequence physicochemical properties.

## 3. Conclusions

The ECM offers a complex environment to sustain cell growth and differentiation that over the last few decades scientists began to unravel, for applications in regenerative medicine. In particular, we have witnessed a plethora of studies that confirmed short, self-assembling peptides as ideal building blocks to mimic the structural features of the ECM nanofibrous hydrogel. In parallel, several scientists have been decoding the minimalistic peptide sequences of the ECM components that bear bioactivity, often through integrin-engagement to promote cell adhesion and migration. As the two fields keep advancing, in the last 5 years we have been witnessing the combination of self-assembling and bioactive motifs into functional hydrogel scaffolds, through covalent and non-covalent approaches, also in some cases exploiting enzymes as triggers for assembly and/or disassembly.

The multi-component approach is promising not only to better recapitulate the complexity of the ECM, e.g., through the inclusion of both peptides and polysaccharides, but also to offer the means to fine-tune the bioactive, gelling, and viscoelastic properties of the systems. Indeed, a key challenge that is often encountered as mentioned in this Review, is the fact that bioactive sequences are often hydrophilic and present ionizable groups that can interfere with the hydrophobically-driven self-assembly of short peptides. To this end, a successful approach has been the combination of positively and negatively charged sequences through co-assembly promoted by electrostatic interactions, or the careful design of peptide sequences with additional amino acids to better control the overall charge and self-assembly at physiological pH.

**Figure 5:**
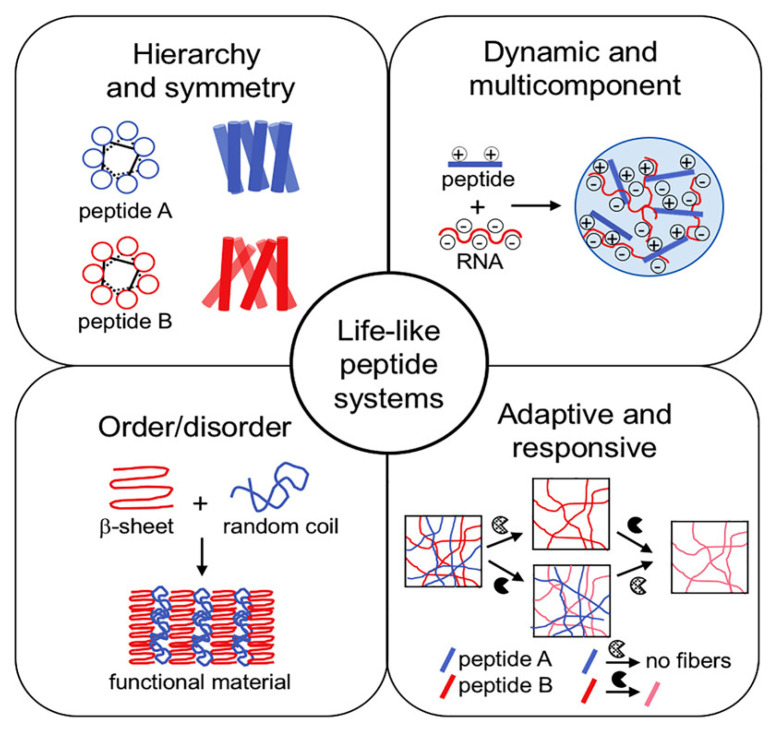
Life-like systems are out-of-equilibrium and present several useful features to mimic living tissues. Reproduced from [131], copyright © 2022, with permission from Elsevier.

Despite the many successes, clearly, the ECM mimetics available today are still rudimental, when compared to the natural ECM fine complexity and dynamism. In particular, recent studies have revealed how not only the surface availability, but also the mobility, of bioactive motifs is key to attaining the desired effects [81]. Other studies are pointing to out-of-equilibrium self-assembled hydrogels to better mimic the dynamic nature of natural tissues, together with elements of hierarchical assembly, order/disorder, and so on (Figure 5) [131]. Furthermore, the inclusion of different types of biomolecules offers net advantages in terms of biomaterial performance relative to peptide-only systems, as we have mentioned in this review. Lastly, the next-generation scaffolds need to feature structural elements to increase their lifetime and slow down the biodegradation rate by enzymes. Different avenues are possible to this end, spanning from the inclusion of d-amino acids [132] or non-natural amino acids [133], to chemical crosslinking ideally with biocompatible agents [134]. Now is the time to raise the bar for the biomaterials scientists and take on the challenge to combine together all these elements, as well as structural features to slow down biodegradation rates by endogenous enzymes, to realize the awaited promise of full repair of human tissues through fine-level regenerative medicine.

## Data Availability

Not applicable.
